# Determinants of anthropometric characteristics of under-five children in internally displaced persons´ camps in Abuja municipal area council, Abuja, Nigeria

**DOI:** 10.11604/pamj.2020.36.313.21221

**Published:** 2020-08-20

**Authors:** Olufunminiyi Samson Idowu, Abimbola Ellen Akindolire, Bosede Ehelamioke Adebayo, Ayodeji Matthew Adebayo, Oluwaseun Ariyo

**Affiliations:** 1 Department of Community Medicine, University College Hospital, Ibadan, Nigeria,; 2 Department of Paediatrics, College of Medicine, University of Ibadan, Ibadan, Nigeria,; 3 Department of Community Medicine, College of Medicine, University of Ibadan, Ibadan, Nigeria,; 4 Department of Human Nutrition, College of Medicine, University of Ibadan, Ibadan, Nigeria

**Keywords:** Stunting, underweight, wasting, predictors, under-five children, internally displaced persons, child malnutrition, infant mortality, immunization, Nigeria

## Abstract

**Introduction:**

displacement predisposes to deprivation and hunger and consequently malnutrition. In Nigeria, information on anthropometric characteristics and associated factors among displaced under-five children is important to strengthen strategies to ameliorate malnutrition and promote child health. This study was conducted to identify the determinants on anthropometric indices among under-five children in internally displaced persons’ camps in Abuja, Nigeria.

**Methods:**

this cross-sectional study involved 317 mother-child (0-59 months) pairs selected using two-stage simple random sampling technique. Information on socio-demographic, care practices (infant feeding, immunization, deworming) and anthropometric characteristics of index children was obtained using semi-structured, interviewer-administered questionnaire. Weight and length/height were assessed using standard procedure and analysed using World Health Organization (WHO) Anthro software. Data were analysed using descriptive statistics and logistic regression at p<0.05.

**Results:**

median age was 24 months, 50.8% were male and 42.3% were delivered at health facility. Only 45.4% were exclusively breastfed, 28.8% were fed complementary foods too early, 45.4% were dewormed in the preceding six months and 43.9% had complete/up-to-date immunisation. Prevalence of underweight, stunting and wasting was 42%, 41% and 29.3%, respectively. Poor anthropometric indices were higher among male than female children, except wasting. Having good anthropometric index was 2.5 times higher among children <12 months than children ≥37 months (CI: 1.08-5.8), 2.4 times higher among 1^st^ birth order than 5^th^ orders (CI: 0.19-0.93), 1.7 times higher among female than male children (CI: 1.08-2.82).

**Conclusion:**

malnutrition is a major health problem among under-five children in internally displaced camps and major determinants include age, birth order, gender and deworming status.

## Introduction

In the last few decades, wars, conflicts, insurgency and displacement of people have contributed significantly to the rising burden of malnutrition in many parts of Africa including Nigeria. Currently, Nigeria has about three million internally displaced persons (IDPs) and ranked first in sub-Saharan Africa and third on the global scale (behind Syria of 6.5 million and Colombia of 5.7 million) [1]. Presently, IDPs in Nigeria consist of 352,840 households distributed across 22 states [2] and children under-five years of age account for about 53.7% of this population [2]. These children are at a period of rapid physical and mental development greatly influenced by nutrition among other environmental factors. Malnutrition among under-five children remains a global challenge especially in the low- and middle-income countries. As at 2018, the global reports showed little improvement in nutrition situation among children as stunting and wasting affect 149 million (21.9%) and 49.5 million (7.3%) children, respectively compared to 156 million (23.2%) and 50 million (7.4%) in 2016 [3]. Presently, about 60 million and 12 million under-five children in Africa suffer from stunting and wasting [3], respectively, out of which, Nigerian children constitute about 12 and 2.2 million, respectively [4]. This suggest that Nigeria singly contributed about 20 and 18 percent to the burden of stunting and wasting in Africa. According to the 2018 Nigeria Demographic and Health Survey (NDHS), about 37% of under-fives were stunted, 7.1% wasted and 22.7% underweight [5].

The report reflects a marginal decline in the burden of underweight and wasting while prevalence of stunting remains high. Likewise, the north-south divide in the prevalence of malnutrition remains. The perpetuity of high burden of malnutrition in northern Nigeria requires a concerted effort to promote health and well-being of the large number of children in these regions and also identify the burden of malnutrition among displaced people to delineate the contribution of displacement to the burden of malnutrition in the country. The high burden of malnutrition in northern Nigeria has been attributed to several factors including low socioeconomic status and poor education of parents, especially mothers [6] and increasing ethnic, religious and political conflicts [7,8]. These factors are in addition to numerous factors including poor nutrition knowledge, poor infant and young child feeding practices and poor care practices that generally undermine nutrition situation in Nigeria [5]. Furthermore, under-five children in IDP camps are at risk of malnutrition following several limiting factors including, poor infant and young child feeding support, low birth interval between the children, poor infants and young child caring practices and inadequate coordination of humanitarian assistance [9,10]. According to Guerrier, the prevalence of malnutrition among under-five children in various IDP camps across Africa ranged from 6% to as high as 52% [11].

Malnutrition is a threat to health and well-being of children and mortality related to malnutrition in IDP camps causes about 17% of total death among the under-five children in Kenya [12]. The nutritional status of under-fives in Nigeria across different categories and regions have been studied and documented by various researchers, however, little is known about anthropometric characteristics of children under special social conditions such as internal displacement and its determining factors. The poor living condition in the IDP camps predisposes victims, especially under-fives, to adverse conditions such as food deprivation and hunger leading to malnutrition. Malnutrition in this group can have devastating long-term effects such as poor cognition and ultimately failure to attain full potential. Also, various interventions by different stakeholders have focused on the needs of the displaced adult men and women neglecting the sensitive needs of under-five children in IDP camps. Similarly, most relief operations from international, national and non-governmental organisation (NGO) stakeholders have concentrated on designated “official camps” such as Maiduguri, Borno state leaving other IDP in so called “non-official camps” like Abuja without humanitarian support and assistance. Hence this study was conducted to assess the determinants of anthropometric characteristics of under-five children in IDP camps in Abuja so as to provide relevant information, which may be needful to identify areas of interventions to ameliorate the adverse effect of poor nutrition on these children.

## Methods

**Study area:** this study was conducted in Abuja, the capital of Nigeria. The city has an altitude of 477mm, average temperature and rainfall of 25.7°C and 1389mm, respectively. The climate is tropical with two main seasons; rainy and dry seasons. The city falls in the middle belt of the country which is characterized with high food production including yam, cassava, cereals and various fruits and vegetables. The study locations are selected IDP camps in the city.

**Study design and population:** a descriptive cross-sectional study was conducted in selected IDP camps. The study population comprised of the mothers or caregivers with children between 0-59 months who have resided in Abuja IDP camps for more than six months and were available in the camp during the period of the study. Mothers or caregivers with under-fives who were visiting the camp at the time of the study were excluded. A minimum sample of 288 was determined using the Leslie Kish formula for estimating minimum sample size for descriptive study assuming standard normal deviate (Zα) at 95% CI of 1.96, prevalence (p) of childhood malnutrition in IDP camps from previous study [13] of 52.4% and 5% level of precision. A two-stage sampling technique was used to recruit study participants into the study. In the first stage, three IDP camps (Durumi, Gurku and Sunshine estate) out of the four IDP camps with the highest number of children in Garki area of Abuja were identified and selected using simple random sampling technique by balloting. In the second stage, mothers/caregivers with children 0-59 months from the three sampled IDP camps were selected using simple random sampling technique. Where more than one child within the ages of 0-59 months was found with the same mother or care giver, simple random technique by balloting was also used to select one of them as study participant and the child selected was referred to as an index child. However, where the index child was of a multiple birth (twin), both children were assessed to conform to the cultural practices.

Data collection: a pretested, semi-structured, interviewer-administered questionnaire was used to obtain information on socio-demographic, care practices, clinical and anthropometric characteristics of the index children. Care practices were evaluated using selected infant feeding practices, immunization and deworming status of the children. Two nurse attendants from Guards Brigade Medical Centre (a military health facility) Abuja who communicate well in both English and Hausa languages were recruited as research assistants and were trained on the method of questionnaire administration. The questionnaire was pre-tested at Karumajiji IDP camp with thirty-two respondents and amendments were made to re-structure the questions that were found ambiguous. The questionnaires were retrieved, collated and sorted out for accuracy and completeness before leaving the field daily. Weight was assessed to the nearest 0.1kg using an electronic body weighing scale. Body height or length (for children below 24 months) was measured to the nearest one centimetre using stadiometer and length board, respectively. All children were dressed in light clothing prior to measurements. Anthropometric indices of sampled children were calculated using reference medians of the World Health Organization (WHO) [14].

Children whose height-for-age (HAZ) -2>SD from the median of the WHO reference population were considered stunted, or chronically malnourished, while -3>SD from the reference median were considered severely stunted. Children with weight- for- height (WHZ) -2>SD were regarded wasted and severely wasted with -3>SD. Those with weight- for- age (WAZ) -2>SD were considered underweight and severely underweight with -3>SD. Data entry and processing were done using IBM Statistical Package for Social Sciences (SPSS) Version. Descriptive statistics are presented in frequencies, proportions and means. Bivariate analysis was conducted at p value of 5% to test for significant associations between socio-demographic variables and anthropometric characteristics. These were loaded to identify determinants of anthropometric characteristics using logistic regression analysis. The study was approved by the department of community medicine, University College Hospital Ibadan. Ethical approval for the study was obtained from the health research ethical committee federal, capital territory Abuja (approval no: FHREC/2017/01/28/04-04-17). Informed consent was obtained from the mothers/caregivers before the procedure.

## Results

A total of three hundred and seventeen under-fives participated in the study. The socio-demographic characteristics of the under-five children are as shown in Table 1. The median age of the children was 24 months (5-48 months) and about half (50.8%) of the children were male. Above half (52.4%) were between 2^nd^ and 4^th^ in birth order, only 22.1% had birth interval of at least 2 years and only 42.3% of the children were delivered at health facility. The care practices for the index children are presented in Table 2. All the children (100.0%) were breastfed, however, 44.5% had water before the recommended age of six months and 45.4% were exclusively breastfed based on mothers/caregivers response. Likewise, 28.8% of the children were fed complementary foods prior to recommended age of six months and only 45.4% were dewormed in the preceding six months. Without immunisation card, 71.3% of the children had complete/up-to-date routine immunisation and when confirmed with immunisation card, 43.9% had complete/up-to-date immunisation. Table 3 shows the anthropometric characteristics of the sampled under-five children. The prevalence of underweight among the under-five children was 42%, out of which about 9% were severely underweight. Gender disaggregation shows higher burden of underweight among male (46.0%) compared to female children (37.8%). Prevalence of stunting was 41%, out of which, 12.9% were severely stunted. Stunting was also higher among male (44.7%) compared to female children (37.2%).

**Table 1:** socio-demographic characteristics of under-five children in IDP camps in Abuja municipal area council

Variable (N=317)	Frequency	%
**Age (months)**		
<12	52	16.4
12-36	199	62.8
≥37	66	20.8
**Gender**		
Male	161	50.8
Female	156	49.2
**Birth order**		
1^st^	60	18.9
2-4	166	52.4
≥5	90	28.5
Non response	1	0.3
**Birth interval**		
1^st^ and only child	60	18.9
<2 years	187	59.0
≥2 years	70	22.1
**Place of delivery**		
Health facility	134	42.3
Home	184	57.7

**Table 2:** care practices for the index children

Variable (N=317)	Frequency	%
Child ever breastfed		
Yes	317	100.0
No	-	-
**Age at which water was introduced**		
Before 6 months	174	55.4
After 6 months	143	44.5
**Exclusively breastfed**		
Yes	142	45.4
No	171	54.6
**Age at which complementary feeding was introduced**		
Before 6 months	91	28.8
After 6 months	226	71.3
**Child dewormed in the preceding 6 months**		
Yes	144	45.4
No	173	54.6
**Immunization card seen**		
Yes	201	63.4
No	116	36.6
**Reported immunisation status**		
None	34	10.7
Up to date	22	6.9
Completed	204	64.4 (*43.9%)
Not completed	57	18.0

* Immunization status based on immunization card

**Table 3:** anthropometric indices of under-five children

	Sex			
	Male n(%)	Female n(%)	Total n(%)	National average (NDHS 2018)
WAZ	161	156	317	
Normal	87(54.0)	97(62.2)	184(58.0)	
Undernutrition	74(46.0)	59(37.8%)	133(42.0)	22.7
Severe undernutrition	16(9.9)	12(7.7)	28(8.8)	8.1
**HAZ**				
Normal	89(55.3)	98(62.8)	187(59.0)	
Stunting	72(44.7)	58(37.2)	130(41.0)	37
Severe stunting	25(15.5)	16(10.3)	41(12.9)	19.1
**WHZ**				
Obese	0	2(1.3)	2(0.6)	
Normal	114(70.8)	108(69.2)	222(70.0)	
Wasting	47(29.2)	46(29.5)	93(29.3)	7.1
Severe wasting	10(6.2)	8(5.1)	18(5.7)	2.0
				

Similarly, prevalence of wasting was 29.3%, out of which 5.3% were severely wasted, however, wasting was higher in female (29.5%) than male children (29.2%). The identified determinants of anthropometric characteristics among under-five children in the sampled IDP camps are as shown in Table 4. The model included age of the children, birth order of the child, history of deworming, immunisation status, gender, morbidity status, fluffy hair and household monthly income. Children who were younger in age (<12 months) are 2.5 times more likely than older children ≥37 months to have good anthropometric index (95% CI 1.078-5.780; p =0.033). Children who were of the 1^st^ birth order are 2.4 times more likely than those in the 5^th^ orders to have good anthropometric index (95% CI=0.191-0.925; p=0.031). Female children are 1.7 times more likely than male to have good anthropometric index (95% CI=1.080-2.816; p=0.023). Dewormed children are 0.5 times less likely than non-dewormed to have good anthropometric index (95% CI=0.317-0.828; p=0.006). Children with no history of morbidity in the last two weeks are 2.9 times more likely than those with morbid conditions to have good anthropometric index (95% CI 1.024-8.537; p=0.045).

**Table 4:** determinants of anthropometric characteristics of under-five children

Variables	Adjusted odds ratio	95%CI	p value
**Age of the child (months)**			
≤12	2.496	1.078-5.780	0.033*
13-36	1.598	0.845-3.021	0.149
≥37 (Ref)	1.000		
**Birth order of the child**			
1^st^	2.398	0.191-0.925	0.031*
2-4	1.592	0.231-1.299	0.547
≥5 (Ref)	1.000		
**Gender of the child**			
Male (Ref)	1.000		
Female	1.744	1.080-2.816	0.023*
**Immunisation status of the child confirmed with card**			
Up to date (Ref)	1.000		
Not completed	2.554	0.727-8.978	0.144
Completed	0.733	0.332-1.620	0.443
Card not seen	1.336	0.776-2.301	0.296
**Deworming practices in the child**			
Yes	0.511	0.317-0.828	0.006*
No (Ref)	1.000		
**Morbidity status**			
Yes (Ref)	1.000		
No	2.961	1.024-8.537	0.045*
**Hair fluffiness in the child**			
Yes (Ref)	1.000		
No	1.442	0.875-2.377	0.151
**Household monthly income**			
<₦10,000.00 (<$31)	1.008	0.574-1.769	0.978
≥₦10,000.00 (≥$31). (Ref)	1.000		
* Significant at P<5%			

## Discussion

Malnutrition is unacceptably high in this study population with at least one-third of the under-five children affected. Studies across Africa have reported a lower burden of malnutrition among children in IDP camps ranging from 13.4-20.9% [12,15,16]. The higher burden in the current study could be a result of inadequate humanitarian assistance or intervention from the national government or international humanitarian agencies in the sampled IDP camps. Reports from IDP camps in Kenya [16] and Sudan [15] showed strongly coordinated humanitarian intervention from both the national government and international organizations which contributed to comparatively low prevalence of malnutrition reported in these countries. An earlier study in Jos, Nigeria has shown that malnutrition is not inevitable in emergency and where adequate intervention or support services are in place, growth failure could be prevented among children in IDP camps [17]. Despite this, prevalence of underweight, stunting and wasting in this study is 42.0%, 41.0% and 29.3%, respectively. Severe underweight, stunting and wasting afflict 8.8%, 12.9% and 5.7% of the under-five children, respectively. Using WHO [18] threshold of public health significance of <10%, <20% and <5% for underweight, stunting and wasting among under-five children, nutrition situation in the study could be considered critical. These under-five children manifest both chronic and acute malnutrition, which portend serious danger to overall well-being. These results reflect higher burden and severity of malnutrition among children in IDP camps compared to the national average.

Underweight is about two times higher than the 22.7% reported among Nigerian under-five children, though the severe underweight is similar [5]. Stunting is also higher than the national average of 37% and wasting is four times higher than national average of 7% [5]. Stunting among the children is similar to 39.9% reported among war torn Afghanistan children by Mashal *et al*. [19], lower than 52.4% among under-five children in Ugandan IDP camps [13] and 54.1% in Mexico [20], but higher than 17.0% among under-five children in Syrian refugee camps [9]. Wasting among the study participants is similar to the 29% prevalence reported by Salama [21] among displaced under-five children in Ethiopia. However, the prevalence is higher than the 20.6% reported by Guerrier *et al*. [11] among IDPs in eastern Chad and 0.6% prevalence among the Syrian IDPs by Hoetjes *et al*. [22]. The increased susceptibility to food insecurity associated with displacement could be responsible for this high burden of underweight. The variation in reported indices of malnutrition in the various studies could be associated with pre-displacement nutritional status of the children, level of humanitarian aids and support received from national governments while the displacement lasted and other morbidity conditions in the displaced camps. Likewise, the similarity in the stunting prevalence in this study and the national average is an indication that the nutritional status of this study population was probably similar to the general nutrition situation among under-five children in Nigeria prior to displacement.

Underweight is a reflection of acute malnutrition and suggest inadequate weight gain or growth failure in recent time. The burden of underweight from this study is at variance with similar studies in Somalian IDP camps where Kinyoki *et al*. [23] reported a higher prevalence of 58% and lower prevalence (34.7%) was reported by Jayatissa *et al*. [24] in Sri Lanka relief camp and Olack *et al*. [25] who reported 11.8% among informal urban settlement of Kenya. The prevalence of obesity in this study is low (0.6%) and agree with low incidence reported by Duru *et al*. [26] among under-five children in a rural village of Imo state, Nigeria. The reported obesity in this study may be due to genetic factor (positive family history of obesity), however the cause was not explored as it was beyond the scope of objective of this study. Other factors that could contribute to poor nutritional status in this study include low practices of exclusive breastfeeding and shorter breastfeeding duration and this agrees with earlier studies that document poor Infant and Young Child Feeding (IYCF) practices contribute to malnutrition among children from displaced households [13,27,28]. Poor IYCF practices could attenuate body immunity and increase susceptibility to infections and reduce care practices. Clinical signs of malnutrition are readily noticeable in the study population in form of fluffy hair and hair discoloration which are suggestive of low-level amino acid and protein malnutrition [28,29].

The psychosocial problems of the setting may contribute to poor maternal care of children due to family disintegration and parental struggle for survival. Other factors for high burden of malnutrition in this study could be due to food shortage and poor dietary diversity or diet quality occasioned by monotony of diet. In Nigeria, attention and resources are focused on IDP camps in northeast Nigeria largely considered “official camps” by the national government, hence activities of various development partners are concentrated in these camps. The high burden of malnutrition in the sampled camps showcase the need to put in place actions to protect and promote optimal growth of children in other camps outside the northeast region of Nigeria. This level of malnutrition predisposes to increased morbidity and mortality among under-five children, hence increasing under-five mortality index in the country. Furthermore, surviving children may suffer poor cognitive development with consequences on school performance, productivity and earnings in later life.

Predictors of nutritional status are children´s age, gender, fluffy hair, deworming history, morbidity status and child´s birth order. In this study, younger children below 12 months were found to exhibit better anthropometric indices than children aged 37 months and older and female children exhibit better anthropometric indices than male children. Disparity in anthropometric characteristics by age among under-five children have been documented by earlier studies in Africa [30,31]. This observation could be attributed to the supportive role of breastmilk and the significance of breastmilk contribution to meeting nutritional requirements in this age category. As age increases and contribution of breastmilk to nutritional requirements decline and dependence of dietary sources become inevitable, anthropometric indices decline. The gender disparity in anthropometric characteristics is a reflection of the general observation among under-five children in sub-Saharan Africa with stunting predominance among male than female children other studies have reported similar finding [32]. The tendency for male children to have poor nutritional status may be associated with higher nutritional requirements following increased physical activity compared with mostly sedentary female counterparts. Consequently, limited resources which affect dietary intake in poor socioeconomic households and displaced population could adversely influence nutrition and health of male children compared to female children.

## Conclusion

Malnutrition remains a major health problem among under-five children in internal displaced camps with prevalence of underweight, stunting and wasting at 42.0%, 41.0% and 29.3%, respectively. Gender disparity in the burden of malnutrition also exists as underweight and stunting were higher among male than female children. The key determinants of malnutrition in this population include age, birth order, gender and deworming status.

### What is known about this topic

- Malnutrition is high among various categories of population in internally displaced camps and children constitute one of the nutritionally vulnerable groups in this population;
- Malnutrition among children results in impaired physical and cognitive development with consequences on health, learning abilities and earnings in later life.

### What this study adds

- This study reveals the burden and severity of malnutrition in this population;
- The study also reveals the gender dynamics in the state of nutrition among the under-five children in internally displaced persons camps in Abuja, Nigeria;
- The study contributed to the existing body of knowledge in this area the key determinants of malnutrition among under-five children in IDP camps.

## References

[1] United Nations Children Fund (2014). Joint nutrition assessment: Syrian refugees in Lebanon. WHO.

[2] National Population Commission (2007). National census, 2006 provisional figures. National Population Commission-Federal Republic of Nigeria.

[3] Govt Nigeria IOM (2016). Displacement Tracking Matrix, DTM, Round IX Report - April 2016. Relief Web.

[4] UNICEF, WHO and WORLD BANK (2016). Levels and trends in child malnutrition. WHO.

[5] National Population Commission (NPC) (Nigeria) and ICF (2018). Nigeria demographic and health survey key indicators report.

[6] Hamidu JL, Salami HA, Ekanem AU, Hamman L (2003). Prevalence of protein-energy malnutrition in Maiduguri, Nigeria. African Journal of Biomedical Research.

[7] Oboh VU, Hyande A, Gyuse TT, Ajene O (2006). Impact of communal conflicts on agricultural production in Oye Community of Oju LGA in Benue State. Conflicts in the Benue Valley, Makurdi 2006.

[8] Kah HK (2017). Boko Haram is losing, but so is food production: conflict and food insecurity in Nigeria and Cameroon. Africa Development.

[9] Bilukha OO, Jayasekaran D, Burton A, Faender G, King Ori J, Amiri M (2014). Nutritional status of women and child refugees from Syria- Jordan 2014. MWR: Morbidity & Mortality Weekly Report.

[10] Sithole WW (2012). A critical analysis of the impact of food aid on internally displaced persons: the case of Manicaland food aid interventions in Zimbabwe MA Dissertation.

[11] Guerrier G, Zounoun M, Delarosa O, Defourny I, Lacharite M, Brown V (2009). Malnutrition and mortality patterns among internally displaced and non displaced population living in a camp, a village or a town in eastern Chad. PLoS ONE.

[12] Feikin DR, Adazu K, Obor D, Ogwang S, Vulule J, Hamel MJ (2010). Mortality and health among internally displaced persons in western Kenya following post-election violence, 2008, Novel use of demographic surveillance. Bulletin of the World Health Organization.

[13] Olwedo MA, Mworozi E, Bachou H, Orach CG (2008). Factors associated with malnutrition among children in internally displaced person´s camps, northern Uganda. African Health Sciences.

[14] World Health Organization (2006). Child growth standards: length/height for age, weight for age, weight for length, weight for height and body mass index for age: methods and development. Geneva: World Health Organization.

[15] Musa TH, Musa HH, Ali EA, Musa NE (2014). Prevalence of malnutrition among children under five years old in Khartoum State, Sudan. Polish Annals of Medicine.

[16] Polonsky JA, Axelle R, Iza C, Monica R, Klaudia P (2013). High levels of mortality, malnutrition and measles, among recently-displaced Somali refugees in Dagahaley camp, Dadaab refugee camp complex, Kenya Conflict and Health. Conflict and Health.

[17] Glew RH, Bhanji RA, VanderJagt DJ (2003). Effects of displacement resulting from ethnic/religious conflict on the growth and body composition of Fulani children in northern Nigeria. Journal of Tropical Pediatrics.

[18] de Onis M, Blössner M (1997). World Health Organization, WHO global database on child growth and malnutrition.

[19] Mashal T, Takano T, Nakamura K, Kizuki M, Hemat S, Watanabe M (2008). Factors associated with the health and nutritional status of children under 5 years of age in Afghanistan: family behaviour related to women and past experience of war-related hardships. BMC Public Health.

[20] Sanchez-PérSáez HJ, Hernán MA, Ríos-González A, Arana-Cedeño M, Navarro A, Ford D (2007). Malnutrition among children younger than 5 years-old in conflict zones of Chiapas, Mexico. American Journal of Public Health.

[21] Salama P, Assefa F, Talley L, Spiegel P, van Der Veen A, Gotway CA (2001). Malnutrition, measles, mortality and the humanitarian response during a famine in Ethiopia. JAMA.

[22] Hoetjes BM, Rhymer W, Matasci-phelippeau L, van der Kam S (2015). Emerging cases of malnutrition amongst IDPs in Tal Abyad district, Syria MSF operations in Northern Syria. Field Exchange.

[23] Kinyoki DK, Moloney GM, Uthman OA, Kandala N, Odundo EO, Noor AM (2017). Conflict in Somalia: impact on child undernutrition. BMJ Glob Health.

[24] Jayatissa R, Bekele A, Piyasena CL, Mahamithawa S (2006). Assessment of nutritional status of children under five years of age, pregnant women and lactating women living in relief camps after the tsunami in Sri Lanka. Food and Nutrition Bulletin.

[25] Olack B, Burke H, Cosmas L, Bamrah S, Dooling K, Feikin DR (2011). Nutritional status of under-five children living in an informal urban settlement in Nairobi, Kenya. J Health Popul Nutr.

[26] Duru CB, Uwakwe KA, Oluoha UR, Nnebue CC, Achigbu KI, Diwe JV (2015). Anthropometry and nutritional status of pre-school children in a rural community in the Niger Delta Region of Nigeria. Int J Curr Res Biosci Plant Biol.

[27] Zakanj Z, Armano G, Grguric J, Herceg-Cavrak V (2000). Influence of 1991-1995 war on breast-feeding in Croatia: Questionnaire study. Croatian Medical Journal.

[28] Jakobsen M, Sodemann M, Nylén G, Balé C, Nielsen J, Lisse I (2003). Breastfeeding status as a predictor of mortality among refugee children in an emergency situation in Guinea Bissau. Tropical Medicine and International Health.

[29] Goldberg LJ, Lenzy Y (2010). Nutrition and hair. Clinics in Dermatology.

[30] Abubakar A, Uriyo J, Msuya SE, Swai M, Stray-Pedesen B (2012). Prevalence and risk factors for poor nutritional status among children in the Kilimanjaro region of Tanzania. Int J Environ Res Public Health.

[31] Endris N, Asefa H, Dube L (2017). Prevalence of malnutrition and associated factors among children in rural Ethiopia. Hindawi Biomed Research International.

[32] Wamani H, Astrøm AN, Peterson S, Tumwine JK, Tylleskar T (2007). Boys are more stunted than girls in sub-Saharan Africa: a meta-analysis of 16 demographic and health surveys, 10, 110. BMC Paediatrics.

